# Estimation of Minimum Biofilm Eradication Concentration (MBEC) on *In Vivo* Biofilm on Orthopedic Implants in a Rodent Femoral Infection Model

**DOI:** 10.3389/fcimb.2022.896978

**Published:** 2022-07-01

**Authors:** Yu Okae, Kohei Nishitani, Akio Sakamoto, Toshiyuki Kawai, Takuya Tomizawa, Motoo Saito, Yutaka Kuroda, Shuichi Matsuda

**Affiliations:** Department of Orthopaedic Surgery, Graduate School of Medicine, Kyoto University, Kyoto, Japan

**Keywords:** antibiotics, biofilm, infection, implant, *Staphylococcus aureus -* bacteria

## Abstract

The formation of a biofilm on the implant surface is a major cause of intractable implant-associated infection. To investigate the antibiotic concentration needed to eradicate the bacteria inside a biofilm, the minimum biofilm eradication concentration (MBEC) has been used, mostly against *in vitro* biofilms on plastic surfaces. To produce a more clinically relevant environment, an MBEC assay against biofilms on stainless-steel implants formed in a rat femoral infection model was developed. The rats were implanted with stainless steel screws contaminated by two *Staphylococcus aureus* strains (UAMS-1, methicillin-sensitive *Staphylococcus aureus*; USA300LAC, methicillin-resistant *Staphylococcus aureus*) and euthanized on days 3 and 14. Implants were harvested, washed, and incubated with various concentrations (64–4096 μg/mL) of gentamicin (GM), vancomycin (VA), or cefazolin (CZ) with or without an accompanying systemic treatment dose of VA (20 μg/mL) or rifampicin (RF) (1.5 μg/mL) for 24 h. The implant was vortexed and sonicated, the biofilm was removed, and the implant was re-incubated to determine bacterial recovery. MBEC on the removed biofilm and implant was defined as *in vivo* MBEC and *in vivo* implant MBEC, respectively, and the concentrations of 100% and 60% eradication were defined as MBEC_100_ and MBEC_60_, respectively. As for *in vivo* MBEC, MBEC_100_ of GM was 256–1024 μg/mL, but that of VA and CZ ranged from 2048–4096 μg/mL. Surprisingly, the *in vivo* implant MBEC was much higher, ranging from 2048 μg/mL to more than 4096 μg/mL. The addition of RF, not VA, as a secondary antibiotic was effective, and MBEC_60_ on day 3 USA300LAC biofilm was reduced from 1024 μg/mL with GM alone to 128 μg/mL in combination with RF and the MBEC_60_ on day 14 USA300LAC biofilm was reduced from 2048 μg/mL in GM alone to 256 μg/mL in combination with RF. In conclusion, a novel MBEC assay for *in vivo* biofilms on orthopedic implants was developed. GM was the most effective against both methicillin-sensitive and methicillin-resistant *Staphylococcus aureus*, in *in vivo* biofilms, and the addition of a systemic concentration of RF reduced MBEC of GM. Early initiation of treatment is desired because the required concentration of antibiotics increases with biofilm maturation.

## Introduction

Orthopedic infections remain a major clinical problem, with catastrophic costs to healthcare systems (Castaneda et al., 2016). Infections associated with orthopedic implants continue to be life-threatening and devastating complications after orthopedic surgery. *Staphylococcus aureus* (*S. aureus*) and coagulase-negative staphylococci (CNS) are commonly found bacteria in implant-related infections. Among them, methicillin-resistant *S. aureus* (MRSA) is known to be the most difficult to treat organism. Previous studies have reported a 72% cure rate for methicillin-sensitive *S. aureus* (MSSA) infections, whereas the cure rate dropped to 57% for MRSA (Teterycz et al., 2010).

The formation of biofilms on implants is one of the major reasons for failure in cases of implant-associated infection (Masters et al., 2019; Saeed et al., 2019). Biofilms act as a physical barrier and protect bacteria from attack by the host’s immune cells or antimicrobial agents (Zhang et al., 2020) Bacteria on the surface of implants are hidden within the biofilm, preventing host immune cells and antibiotics from attacking them (Costerton, 2005; Flemming and Wingender, 2010; Masters et al., 2019). In addition, the bacteria present in the biofilm acquire pathogenicity and antimicrobial resistance through their ability to exchange information with each other through the quorum sensing mechanism (Rutherford and Bassler, 2012). Biofilm formation occurs in several stages. These stages, which have been observed in both *in vitro* and *in vivo* biofilms, begin with initial attachment, followed by proliferation, biofilm formation, and spread of the mature biofilm (Costerton et al., 1987; O’Toole et al., 2000; Nishitani et al., 2015). In the very early stages of infection, when the biofilm is immature, it may be possible to deal with an implant-associated infection by the administration of standard systemic antibiotics. However, once the biofilm matures, it is difficult to eradicate bacteria within the biofilm using standard antimicrobial treatment (Tomizawa et al., 2021). A high-dose, local antibiotic regimen may have the potential to treat mature biofilms, but effective antibiotics and their effective concentrations are not known.

The efficacy of antibiotics against bacteria is commonly represented by the use of the minimum inhibitory concentration (MIC). MIC is defined as “the minimum concentration of an antibiotic to inhibit bacterial growth” (Li et al., 2017). MIC is generally ascertained by exposing defined amounts of bacteria to increasing concentrations of an antibiotic. However, the methods to derive the measurement are performed on planktonic bacteria in a growth medium. Most bacteria are in biofilm form in orthopedic infections and not in planktonic form (Stoodley et al., 2011; Li et al., 2017). Currently available antibiotic treatments are based on the MIC against the planktonic form of bacteria, and these concentrations are less effective against bacteria in the biofilm, which needs to be up to 1000 times higher. (Verderosa et al., 2019) Thus, the usefulness of MIC for orthopedic implant-associated infections may be limited.

The concept of the minimum biofilm eradication concentration (MBEC) has been proposed as an antibiotic concentration to eradicate bacteria in biofilms (Evans and Holmes, 1987; Girard et al., 2010; Macià et al., 2014). Although MBEC seemed more useful in clinical practice, there are still questions regarding the most appropriate methodologies to measure MBEC. In most current MBEC assays, biofilms are formed on abiotic surfaces, such as polystyrene microtiter well plates, which likely have a different affinity for the attachment of bacteria from the materials used in orthopedic implants, such as titanium alloy or stainless steel (Ceri et al., 2001). Many studies have investigated the effects of antibiotics on biofilms *in vitro* or *ex vivo*, but no study has investigated the real *in vivo* biofilms formed in host animals (Fujimura et al., 2008; Rose and Poppens, 2009; Zimmerli and Sendi, 2019). Compared to *in vitro* biofilms, *in vivo* biofilms are more complex structures composed of bacterial polysaccharides, proteins, DNA, and host structures such as fibrin, fibrinogen, and collagen (Høiby et al., 2010; Nishitani et al., 2015).

To overcome these issues, this study measured MBEC using several related methodologies, First, MBEC was measured using an *in vitro* biofilm formed on a stainless-steel screw. Furthermore, an ambitious attempt was made to evaluate MBEC on *in vivo* biofilms with orthopedic implants using immature and mature *in vivo* biofilms formed on stainless steel screw implants in a rodent infection model.

A related aim of the study was to investigate the clinical utility of standard of care (SOC) antibiotics on *in vivo* biofilms. To that end, this study investigated the *in vitro* and *in vivo* MBEC of gentamicin (GM), cefazolin (CZ), and vancomycin (VA). A selection of these initial assays was then assessed in combination with a systemic dose of either VA or rifampicin (RF) to reflect the usual clinical practice of accompanying local antibiotic therapy with systemic antibiotics.

The purposes of this study were to establish an MBEC assay for orthopedic implants, with a special focus on using *in vivo* biofilms formed in rodents, and to investigate the utility of standard of care (SOC) antibiotics on *in vivo* biofilms.

## Materials and Methods

Two *S. aureus* strains were used in this study. One was UAMS-1, which is a widely used methicillin-sensitive *S. aureu*s (MSSA) strain isolated from an osteomyelitis patient (Gillaspy et al., 1995), and the other was USA300LAC, which is one of the most prevalent and virulent methicillin-resistant *S. aureu*s (MRSA) strains (Kourbatova et al., 2005). All strains were cultured in a tryptic soy broth (TSB) medium. MIC of two strains was determined by measuring the lowest concentration of antibiotics which prevents visible growth of *S. aureus* on the TSB agar plate.

### *In Vitro* MBEC Assay

**Figure 1:**
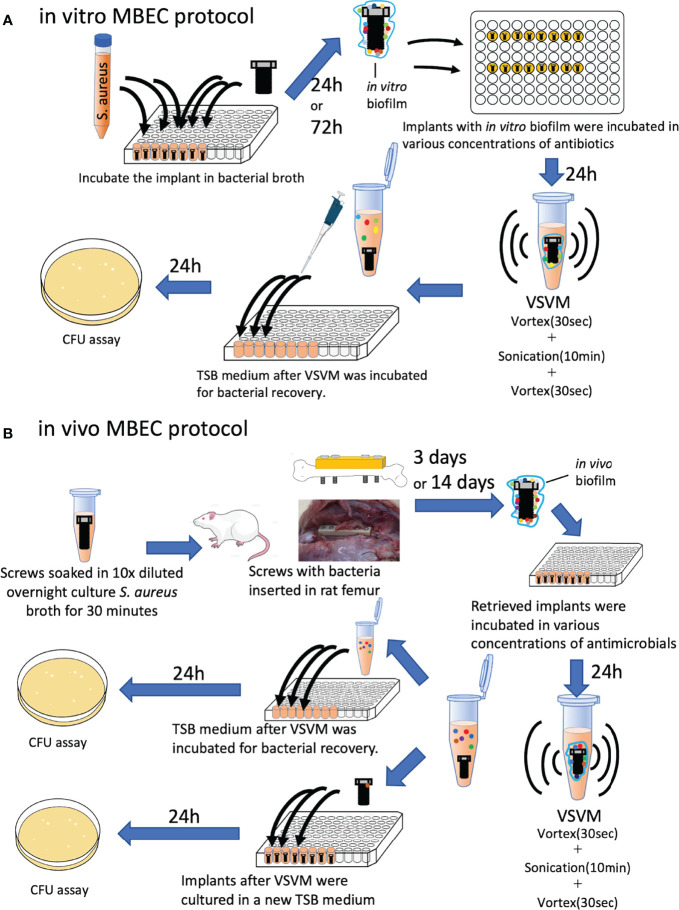
Schematic protocols of **(A)** *in vitro* and *in vivo* **(B)** MBEC assay in this study. *S. aureus*: *Staphylococcus aureus*, VSVM, Vortex-Sonication-Vortex Method, CFU, colony-forming unit.

To evaluate MBEC in orthopedic implants, the MBEC assay was replicated using a stainless-steel screw implant (**Figure 1A**). Sterilized stainless-steel screws (1.2 mm × 8 mm, Esco, Osaka, Japan) were incubated in 250 mL of TSB with *S. aureus* for 24 h (1 day) or 72 h (3 days) shaking at 150 rpm on 96-well polystyrene flat-bottom microtiter plates (Corning, REF 3596, Corning, NY, USA) to allow bacteria to form a biofilm on the implant. The implants were taken out and washed twice with phosphate buffer saline (PBS) to remove any unattached bacteria. The implants were again placed in microtiter plates and treated with various concentrations of antimicrobial agents (GM, VA, and CZ) with or without a systemic treatment dose of VA (20 μg/mL) or RF (1.5 μg/mL) and shaken at 150 rpm at 37°C for 24 h. After 24 h, the implants were removed and placed in a 1.5 mL Eppendorf tube containing fresh TSB solution, and the biofilm adhering to the implants was removed using the Vortex-Sonication-Vortex Method (VSVM) (Oliva et al., 2013; Kırmusaoğlu, 2019). The TSB supernatant was then replaced in a microtiter plate and shaken at 150 rpm at 37°C for 24 h. The recovery of *S. aureus* was quantitatively analyzed by the colony-forming units (CFU) assay by counting the colony number on TSB agar plates. The MBEC in this experiment was defined as an *in vitro* MBEC.

### *In Vivo* MBEC Assay

All animal experiments were conducted with the approval of the university’s animal resources committee and in accordance with national guidelines. Twenty-week-old male Wistar rats were purchased from Japan SLC (Hamamatsu, Japan) and acclimatized to the new environment for 1 week before the interventions. Two rats were housed in one cage with free access to water and standard rodent chow. A constant 12-h light/dark cycle was maintained. After sterilization, the screws were immersed in a 10-fold dilution of an overnight *S. aureus* culture for 30 min. The inoculation load was 6× 10^3^ colony forming units (CFU)/implant. Rats were intraperitoneally administered a combination anesthetic containing 0.375 mg/kg medetomidine, 2 mg/kg midazolam, and 2.5 mg/kg butorphanol to induce anesthesia, which was maintained with 3% isoflurane. Approaches and plating were based on the techniques described in previous reports (Poser et al., 2014; Kawai et al., 2021). After iodine disinfection, a 4-cm skin incision was made on the left lateral thigh and the femur was exposed. A polyetheretherketone plate (4-mm wide, 24-mm long, 2-mm thick with six 1.0-mm screw holes arranged in a line along its length, Umihira, Kyoto, Japan) was placed on the femur, and the femur was drilled with a 1.0 mm drill using the two most proximal and distal holes in the plate. After tapping, four contaminated screws were inserted into the drilled holes. Buprenorphine (0.02 mg/kg) was administered until day 2. After 3 and 14 days, the rats were euthanized by carbon dioxide overdose, and implants with *in vivo* biofilms were harvested (**Figure 1B**). The implants were washed twice with PBS to remove additional bacteria and extra soft tissues, and screws were incubated with various concentrations of antimicrobial agents (GM, VA, and CZ) with or without a systemic treatment dose of VA (20 μg/mL) or RF (1.5 μg/mL) for 24 h shaking at 150 rpm at 37°C. As previously described, the biofilm adhering to the implants was removed using the VSVM. The TSB supernatant was then replaced in a microtiter plate and shaken at 150 rpm at 37°C for 24 h. The recovery of *S. aureus* was quantitatively analyzed using the CFU assay, and the MBEC in this experiment was defined as the *in vivo* MBEC.

As an additional experiment, implants after VSVM were incubated in TSB for 24 h, and CFU recovery was examined to take into account the presence of bacteria in the residual biofilm that had not been removed after VSVM. The MBEC in this experiment was defined as the *in vivo* implant MBEC.

### SEM and EDS Analysis for Biofilm

Scanning electron microscopy (SEM) has been shown to be a suitable tool not only to observe the substratum morphology in detail but also to follow bacterial adhesion and biofilm formation. Energy dispersive X-Ray spectroscopy (EDS) has been shown to be a suitable tool for observing mineral structure formation by bacterial and microalgal biofilms growing on the surface (Abed et al., 2012; Gomes and Mergulhão, 2017). For SEM, additional implants were prepared with USA300LAC incubated for 24 h *in vitro* and for 3 or 14 days *in vivo* in the rat femur. With or without antibiotic (GM or GM +RF) treatment, the implants were fixed overnight in 2.5% glutaraldehyde and 4% paraformaldehyde. Biofilms on the implants were evaluated using SEM (JSM-7900F, JEOL, Akishima, Japan) and EDS (JED-2300F, JEOL).

### Statistics

To determine *in vitro* MBEC, five screws were used for each experimental condition, and the antibiotic concentration that achieved 100% eradication of bacteria in the biofilm was defined as MBEC_100_ and the concentration which achieved 60% eradication of bacteria was defined as MBEC_60_. For the *in vivo* experiments, two rats housed in one cage were defined as one set of animals, and the MBEC of each set was determined. Five MBECs were obtained from 10 rats per experimental conditions, and MBEC_100_ and MBEC_60_ were defined as described before. For animal experiments, MBEC among the three antibiotics (GM, VA, and CZ) or the effect of additional antibiotics (no additional antibiotic, VA, RF) were also statistically compared using the Kruskal–Wallis test with Dunn’s multiple comparison, and p < 0.05 was considered significant.

## Results

### *In Vitro* MBEC

The MIC and MBEC of GM, VA, and CZ on bacteria in the *in vitro* biofilm against UAMS-1 and USA300LAC are shown in **Table 1**. The MBEC_100_ values of the three antibiotics were higher than the MIC. For UAMS-1, VA had the lowest MBEC_60_ for day 1 biofilm, whereas GM had the lowest MBEC_100_ for both day 1 and day 3 biofilm. Against USA300LAC, GM had the lowest MBEC_60_. The MBEC_100_ values of GM and VA were similar for day 1, but GM had the lowest MBEC_100_ for day 3. Against both UAMS-1 and USA300LAC, the MBEC_60_ and MBEC_100_ of CZ were the highest.

**Table 1:** MIC and MBEC on bacteria in *in vitro* biofilm formed on a screw (*in vitro* MBEC).

	MIC	Day 1		Day 3	
		MBEC _60_	MBEC_100_	MBEC _60_	MBEC_100_
	**MSSA(UAMS-1)**				
GM	0.5	64	64	128	128
VA	0.5	32	128	128	256
CZ	1.0	256	512	256	512
	**MRSA (USA300LAC)**				
GM	0.5	32	128	64	128
VA	0.5	64	128	128	256
CZ	128	128	256	512	1024

All data are presented in μg/mL. MIC: minimum inhibitory concentration, MBEC, minimum biofilm eradication concentration; GM, Gentamicin; VA, Vancomycin; CZ, Cefazolin.

### *In Vivo* MBEC

**Figure 2:**
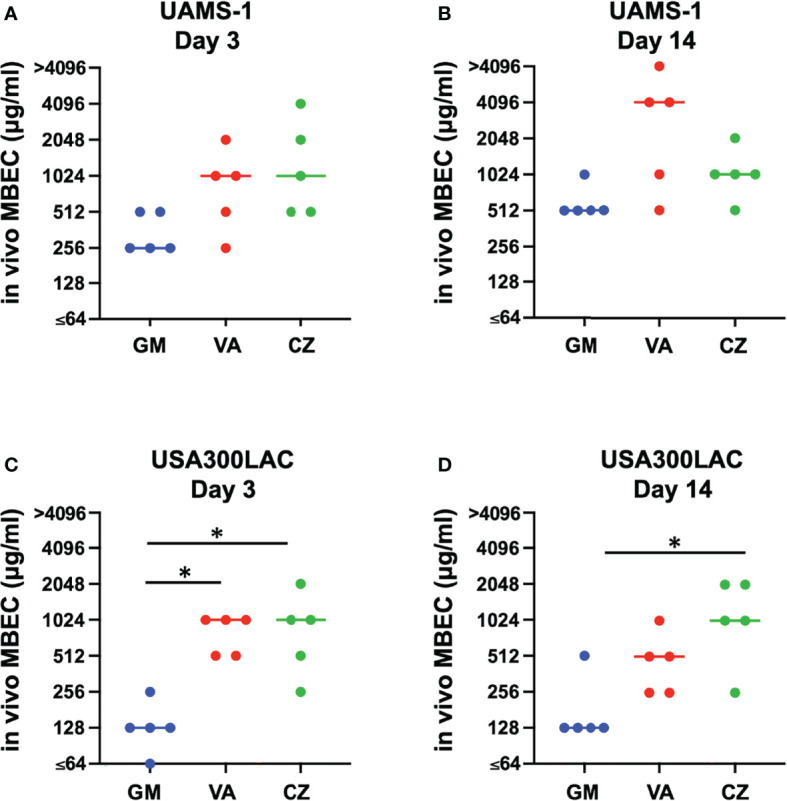
Minimal biofilm eradication concentration of *in vivo* biofilm formed on the implant incubated in rat femur for 3 days and 14 days (*in vivo* MBEC) of UAMS-1 **(A, B)** and USA300LAC **(C, D)**. Bars in each graph indicate the median of five assays. *: p < 0.05 by Kruskal-Wallis test with Dunn’s multiple comparison. GM, gentamicin; VA, vancomycin; CZ, cefazolin.

In this surgical model, infection was limited locally in all rats, and physical debilitation was not observed. No infection-related deaths were observed during the experiment. For UAMS-1, GM had the lowest MBECs among the three antimicrobial agents on both days 3 and 14, although the difference was not statistically significant (**Figures 2A, B**, **Table 2**). A very high concentration of more than 1024 μg/mL was required for MBEC_60_ and MBEC_100_ for VA and CZ. For GM and VA, with the maturation of the biofilm by day 14, MBEC_100_ tended to increase. Similarly, in the USA300LAC group, GM was the most effective among the three antimicrobial agents on both days 3 and 14, with statistical significance (**Figures 2C, D**, **Table 2**). In the case of GM, the longer the implant was incubated, the higher the MBEC_100_. However, MBEC_100_ for VA and CZ did not differ between days 3 and 14.

**Table 2:** MBEC on bacteria on *in vivo* biofilm formed on a screw (*in vivo* MBEC).


	Day 3		Day 14	
	MBEC _60_	MBEC_100_	MBEC _60_	MBEC_100_
**MSSA(UAMS-1)**				
GM	256	512	512	1024
VA	1024	2048	4096	>4096
CZ	1024	4096	1024	2048
**MRSA (USA300LAC)**				
GM	128	256	128	512
VA	1024	1024	512	1024
CZ	1024	2048	1024	2048

All data are presented in μg/mL. MBEC, minimum biofilm eradication concentration; GM, Gentamicin; VA, Vancomycin; CZ, Cefazolin.

### SEM Analysis for *In Vivo* Biofilm

**Figure 3:**
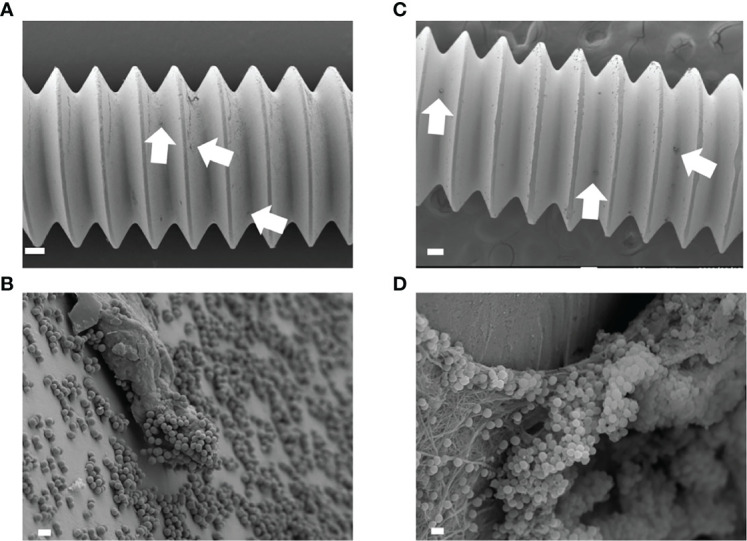
Scanning electron microscopy (SEM) image of USA300LAC infected implant of *in vitro* day 1 **(A, B)** and *in vivo* day 3 **(C, D)**. In low magnification images, a small amount of biofilm formations were observed (white arrow) **(A, C)**. White bars in **A, C** indicate 100 µm, and white bars in **B, D** indicate 1 µm.

**Figure 4:**
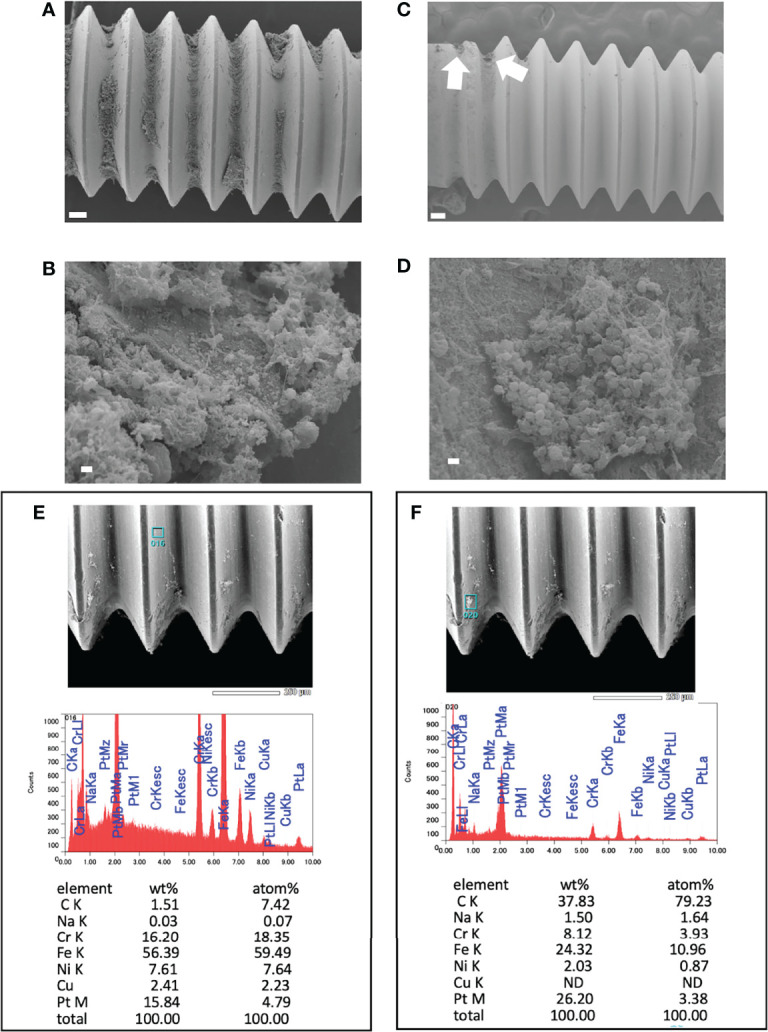
Scanning electron microscopy (SEM) image of USA300LAC infected implant of day 14 *in vivo* biofilm. **(A, B)** are images after 24 h of antibiotics treatment with 512 µg/mL gentamicin which showed robust biofilm formation in the screw grooves. **(C, D)** are images after vortex-sonication-vortex method (VSVM) which has a fare less biofilm compared to before VSVM **(A)**, but a small amount of residual biofilm was observed (white arrows) which has live bacteria **(D)** in the matrix. Energy Dispersive X-Ray Spectroscopy (EDS) on the implant surface **(E)** and on the residual biofilm **(F)** after VSVM was shown with the percentage of containing elements. White bars in A and C indicate 100 μm, and white bars in B and D indicate 1 μm.

A small amount of biofilm was observed in *in vitro* day 1 and *in vivo* day 3 infection with USA300LAC (**Figures 3A, C**). Although simple matrix formation surrounded by bacterial clusters was observed on the *in vitro* implant, bacterial clusters were entangled in a matrix with fiber-like structures on the *in vivo* implant (**Figures 3B, D**). Compared to day 3, a considerable amount of biofilm was observed in the screw groove after 14 days of infection with USA300LAC (**Figures 4A, B**). Even after GM treatment and VSVM, residual biofilm was still observed on the implant with live bacteria (**Figures 4C, D**). EDS showed that residual tissue on the implant after VSVM was not metallic material but tissue from an organism containing much more carbon and less iron and chrome (**Figures 4E, F**).

### *In Vivo* Implant MBEC on Residual Biofilm

**Figure 5:**
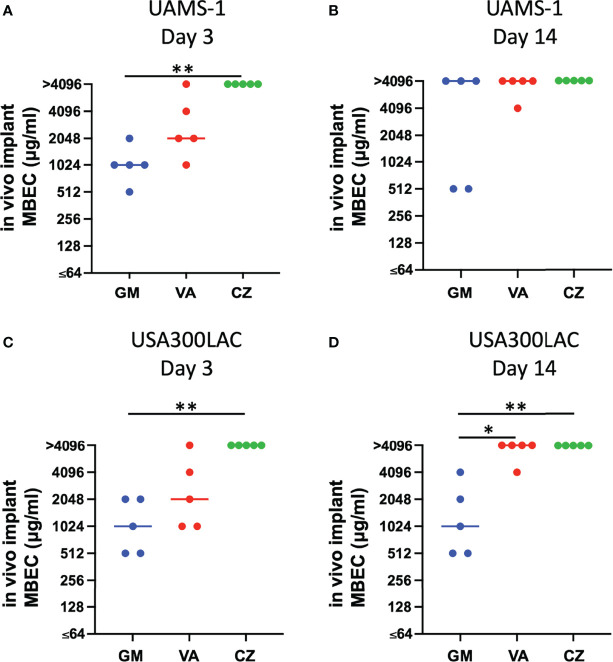
Minimal biofilm eradication concentration against residual *in vivo* biofilm after vortex-sonication-vortex-method. (*in vivo* implant MBEC) of UAMS-1 **(A, B)** and USA300LAC **(C, D)**. Bars in each graph indicate the median of five assays. **: p < 0.01 and *: p < 0.05 by Kruskal–Wallis test with Dunn’s multiple comparison. GM, gentamicin; VA, vancomycin; CZ, cefazolin.

Because residual biofilm was confirmed on the implant after VSVM, bacterial recovery from the residual biofilm was also investigated. Surprisingly, MBECs for bacteria in residual biofilm were far higher than *in vivo* MBEC (**Figure 5**, **Table 3**). Although GM completely eradicated bacteria in the residual biofilm at 2048 μg/mL and 4096 μg/mL for USA300LAC, the other two antibiotics for USA300LAC and all three antibiotics for UAMS-1 on day 14 did not eradicate bacteria completely in the biofilm even with 4096 μg/mL.

**Table 3:** MBEC on residual bacteria in *in vivo* biofilm on a screw after VSVM (*in vivo* implant MBEC).

	Day 3		Day 14	
	MBEC _60_	MBEC_100_	MBEC _60_	MBEC_100_
**MSSA(UAMS-1)**				
GM	1024	2048	>4096	>4096
VA	2048	>4096	>4096	>4096
CZ	>4096	>4096	>4096	>4096
**MRSA(USA300LAC)**				
GM	1024	2048	1024	4096
VA	2048	>4096	>4096	>4096
CZ	>4096	>4096	>4096	>4096

All data are presented in μg/ml. MBEC, minimum biofilm eradication concentration; VSVM, Vortex-Sonication-Vortex Method; GM, Gentamicin; VA, Vancomycin; CZ, Cefazolin.

### Additional Effect of Second Antibiotics

**Figure 6:**
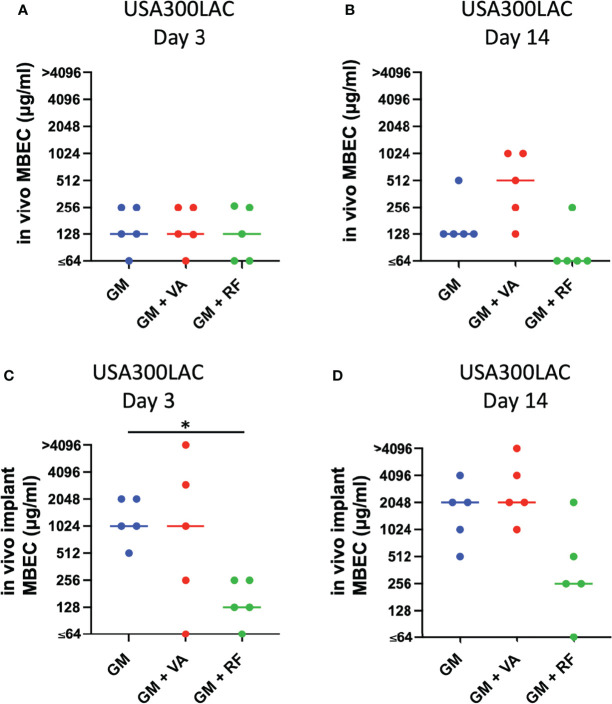
Effect of secondary antibiotics (VA: vancomycin, RF: rifampin) on *in vivo* minimal biofilm eradication concentration (MBEC) of GM (gentamicin) **(A, B)** and against residual *in vivo* biofilm after vortex-sonication-vortex-method on *in vivo* implant (*in vivo* implant MBEC) in combination with GM (gentamicin) **(C, D)** Bars in each graph indicate the median of five assays. *: p < 0.05 by Kruskal–Wallis test with Dunn’s multiple comparison.

**Figure 7:**
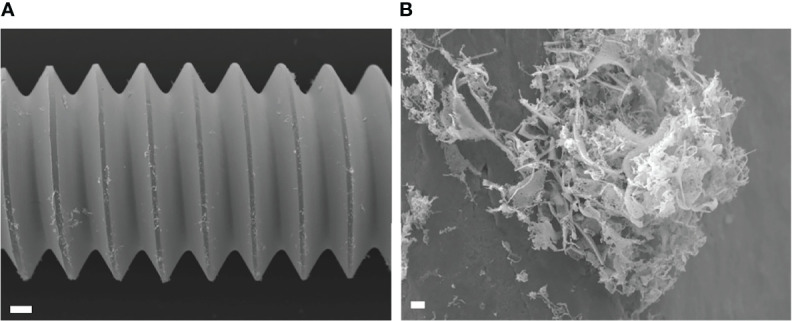
Scanning electron microscopy (SEM) image of USA300LAC infected implant of day 14 *in vivo* biofilm which was treated with 512 µm/mL gentamicin and rifampicin after vortex-sonication-vortex method. Although some residual tissues remained on the implant surface **(A)**, only debris of fibrous matrix without live bacteria in it **(B)**. White bars in A and B indicate 100 μm and 1 μm, respectively.

Local antibiotics are always administered along with systemic antibiotics. Since GM seemed to be the most effective among the three antibiotics for local administration, the additional effect of RF and VA with a systemic concentration on the MBEC of GM was investigated with USA300LAC. The addition of RF reduced the *in vitro* MBEC_60_ and MBEC_100_ of GM in both day 1 and day 3 (**Table 4**). As for *in vivo* MBEC, the addition of systemic doses of RF reduced the MBEC_60_ and MBEC_100_ of GM on day 14 biofilms (**Table 5**), but without a statistical difference (**Figures 6A, B**). For the residual biofilm on the implant after VSVM, although a very high concentration was still needed, RF reduced MBEC_60_ and MBEC_100_ of GM on both day 3 and day 14 biofilms (**Table 6**), with a significant difference on day 3 (**Figures 6C, D**). In contrast to RF, the addition of a systemic dose of VA had no effect on the MBEC of GM in all experiments. SEM showed very little residual biofilm and debris from the fibrous matrix from the implant treated with 512 GM μg/mL plus 1.5μg/mL RF (**Figure 7**).

**Table 4:** Additional effect of second antibiotics in systemic dose on *in vitro* MBEC of GM.

	MRSA(USA300LAC)			
	Day 1		Day 3	
	MBEC _60_	MBEC_100_	MBEC _60_	MBEC_100_
GM	32	128	64	128
GM+VA20μg/ml	128	512	128	128
GM+RF1.5μg/ml	≤4	≤4	64	64

All data are presented in μg/ml. MBEC, minimum biofilm eradication concentration; GM, Gentamicin; VA, Vancomycin; RF, rifampicin.

**Table 5:** Additional effect of second antibiotics in systemic dose on *in vivo* MBEC of GM.

	MRSA(USA300LAC)			
	Day 3		Day 14	
	MBEC _60_	MBEC _100_	MBEC _60_	MBEC _100_
GM	128	256	128	512
GM+VA20 μg/mL	128	256	512	1024
GM+RF1.5 μg/mL	128	256	≤64	256

All data are presented in μg/mL. MBEC, minimum biofilm eradication concentration; GM, Gentamicin; VA, Vancomycin; RF, rifampicin.

**Table 6:** Additional effect of second antibiotics on MBEC of residual bacteria in *in vivo* biofilm on screw after VSVM (*in vivo* implant MBEC) of GM.

	MRSA(USA300LAC)			
	Day3		Day14	
	MBEC _60_	MBEC_100_	MBEC _60_	MBEC_100_
GM	1024	2048	2048	4096
GM+VA 20 μg/mL	1024	>4096	2048	>4096
GM+RF1.5 μg/mL	128	256	256	2048

All data are presented in μg/mL. MBEC, minimum biofilm eradication concentration; VSVM, Vortex-Sonication-Vortex Method; GM, Gentamicin; VA, Vancomycin; RF, rifampicin.

## Discussion

In this study, the potential of SOC antibiotics to eradicate biofilm bacteria was evaluated. Notably, *in vivo* biofilms, which are far more resistant to antibiotics than *in vitro* biofilms, were formed on the implant in the rodent femur and utilized in this study. As expected, the *in vivo* MBEC was higher than the *in vitro* MBEC on stainless steel implants. Not surprisingly, although *in vitro* MBEC ranged from 64–1024 μg/mL, the *in vivo* MBEC was as high as 512–4096 μg/mL. When the implant was incubated for the same duration (3 days), bacteria in the *in vivo* biofilm were more resistant to antibiotics than in the *in vitro* biofilm. SEM analyses showed that remnants of the biofilm were firmly attached to the implant, and far higher concentrations were needed to eradicate bacteria from the residual biofilm. Among the three antibiotics, only GM had the potential to eradicate USA300LAC on day 14 biofilms in 60% of samples at 1024 μg/mL. This high concentration might be possible with a radical local administration, but cytotoxicity is a concern. A combination of systemic doses of second antibiotics was attempted, and the concentration of GM to obtain 60% effectivity was reduced to 256 μg/mL with RF 1.5 μg/mL in day 14 biofilms of USA300LAC.

We also expected that the MBEC would increase as the biofilm matured; thus, MBEC for day 14 biofilms was expected to be much higher than MBEC for day 3 biofilms, because biofilm maturation is reported at 14 days post-infection (Nishitani et al., 2015). Although this trend was confirmed in most cases, within the *in vivo* biofilms detailed in **Table 2**, GM used on USA300LAC showed the same MBEC_60_ on days 3 and 14, and CZ used on UAMS-1 had a lower MBEC_100_ on day 14 than it did on day 3. It was speculated that mature biofilms were firmly attached to the implant surface and difficult to remove in these cases. Although VSVM is an established method for removing biofilms, all the biofilms cannot be removed, and some biofilm remains on the implant (Rosa et al., 2019). In the present study, the presence of residual biofilm was confirmed using SEM after VSVM. Therefore, if antimicrobial agents do not penetrate deep into the biofilm, surviving bacteria may multiply again in the residual biofilm. To this end, the implants were cultured again for 24 h after VSVM to examine biofilm persistence. It is noteworthy that persistent biofilms on the implant surface needed a much higher concentration to eradicate the bacteria inside. This can be demonstrated by the finding that VA and CZ were almost ineffective at experimental concentrations of up to 4096 μg/mL in this study. GM was the most effective, but its MBEC_100_ on the residual biofilm was also very high at 2048 μg/mL for day 3 and 4096 μg/mL for day 14 biofilms. Even with the local administration of antibiotics, it seems difficult to reach these high concentrations in actual clinical practice. Therefore, a combination of antibiotics was considered, as a reduction of MBEC was reported by combining multiple antibiotics in previous studies (Deresinski, 2009) (Dall et al., 2018).

We used the concentration of a systemic administration dose to mimic the condition of the combination of local and systemic therapy. As GM had the lowest MBEC among the three antibiotics, the MBEC of GM was evaluated again in combination with a systemic dose of VA and RF. VA and RF were selected because they have been reported to penetrate deep into biofilms (Jacqueline and Caillon, 2014). The addition of a systemic dose of VA did not reduce the MBEC of GM. On the other hand, RF reduced the *in vivo* MBEC, especially in the *in vivo* implant MBEC on day 3. The reason for this may be that the bactericidal effect of VA in biofilms is inferior to that of RF and RF is reported to be more effective than VA in MRSA biofilm infections) (Jørgensen et al., 2016). Some *in vivo* animal studies have demonstrated the benefits of combined treatment with RF, and these studies have reported that the addition of RF significantly reduces CFU (Stavrakis et al., 2014; Thompson et al., 2017). Previous studies have also reported that sublethal doses of VA induce robust biofilm formation through the promotion of extracellular DNA-dependent release (Hsu et al., 2011; Abdelhady et al., 2014). The 20 μg/mL dose used in this study may be insufficient for bacteria in biofilms, but higher blood concentrations increase the risk of side effects. GM is effective in eradicating biofilms formed by *S. aureus*, including MRSA, and GM’s MBEC has been reported to be lower than that of VA (Mottola et al., 2016; Metsemakers et al., 2018). In addition, the synergistic effect of GM with other antimicrobial agents on staphylococcal biofilms has been reported previously (Dall et al., 2018). GM has a long history of local administration in the form of antibiotic-loaded cements (Vugt et al., 2019), and is also used for systemic administration by intramedullary antibiotic perfusion (Maruo et al., 2021). GM seemed a good candidate for local antibiotic treatment.

This study had some limitations. First, only one bacterial strain of MSSA and MRSA was investigated. Although these bacteria are representative strains that produce robust biofilms (Nishitani et al., 2015), the effectiveness of antibiotics against other *S. aureus* strains remains unknown. GM was the most effective among the three SOC antibiotics, and whether GM has similar efficiency against GM-resistant MRSA strains is unknown. Moreover, other bacterial strains that cause implant-associated infections have not yet been investigated. However, since we established a novel method to investigate *in vivo* MBEC with real implants, it became possible to investigate *in vivo* MBEC with various clinically acquired bacterial strains from patients with implant-associated infections. Second, three SOC antibiotics were selected, but there are many candidate antibiotics, including other anti-MRSA drugs (e.g., linezolid and daptomycin), that might be more effective. Third, stainless steel was used to grow the biofilm, but the MBEC of other implant materials, such as titanium alloy or cobalt-chrome, may be different. Fourth, antibiotic treatment was administered for only 24 h. Some reports have shown that a longer treatment duration reduced the required MBEC (Castaneda et al., 2016; Post et al., 2017). Although very high concentrations were needed for *in vivo* implant MBEC in this study, these high concentrations may not be needed when biofilms on implants are exposed to antibiotics for longer durations. This study only showed the concentration required to eradicate bacteria in biofilms on orthopedic implants within 24 h of treatment.

In conclusion, a novel MBEC assay for *in vivo* biofilms on orthopedic implants was developed. GM was the most effective SOC antibiotic against both MSSA and MRSA biofilms in both the *in vitro* and *in vivo* experiments. Although in this study GM + RF only showed its greater effect against MRSA on day 14, early initiation of treatment with antimicrobial intervention is still desirable in clinical practice because the required concentration of antibiotics increases with the maturation of the biofilm.

## Data Availability Statement

The raw data supporting the conclusions of this article will be made available by the authors, without undue reservation.

## Ethics Statement

The animal study was reviewed and approved by Kyoto University Animal Experiment Committee.

## Author Contributions

KN conceived the ideas for experimental designs, analyzed data, and wrote the manuscript. YO conducted the experiments, analyzed data, and wrote the manuscript. AS and YK acquired findings and advised on interpretations. TK contributed to the development of the animal model. TT and MS conducted the experiments. SM contributed the concept and supervised the project. All authors approved the final version of the manuscript.

## Funding

This study was supported by Grants-in-Aid for Scientific Research (19K09548, 19K09621, 22K09329).

## Conflict of Interest

SM received honoraria for meeting chair and a scholarship donation for orthopaedic research from Diichi-Sankyo, outside of this study. This pharmaceutical company has no contribution or influence on this study.

The remaining authors declare that the research was conducted in the absence of any commercial or financial relationships that could be construed as a potential conflict of interest.

## Publisher’s Note

All claims expressed in this article are solely those of the authors and do not necessarily represent those of their affiliated organizations, or those of the publisher, the editors and the reviewers. Any product that may be evaluated in this article, or claim that may be made by its manufacturer, is not guaranteed or endorsed by the publisher.
